# Bio-Based Admixture (Black Tea Extraction) for Better Performance of Metakaolin Blended Cement Mortars

**DOI:** 10.3390/ma15113994

**Published:** 2022-06-03

**Authors:** Yi Fang, Jialai Wang, Xiaodong Wang, Monica Lages Do Amaral, Hannah Kniffin, Miranda Reed, Liang Wang, Xin Qian

**Affiliations:** 1 Department of Civil, Construction, and Environmental Engineering, The University of Alabama, Tuscaloosa, AL 35487, USA; yfang20@crimson.ua.edu (Y.F.); xwang202@crimson.ua.edu (X.W.); mlagesdoamaral@crimson.ua.edu (M.L.D.A.); hrkniffin@crimson.ua.edu (H.K.); mereed2@crimson.ua.edu (M.R.); 2 School of Civil Engineering and Architecture, Anhui University of Science and Technology, Huainan 232001, China; 2018028@aust.edu.cn; 3 Key Laboratory of Infrastructure Durability and Operation Safety in Airfield of CAAC, Tongji University, Shanghai 201804, China

**Keywords:** black tea extract, metakaolin, mechanical properties, pore structure, nanoindentation, embodied carbon

## Abstract

With high pozzolanic reactivity, metakaolin (MK) is a popular supplementary cementitious material (SCM), which can be used to partially replace Portland cement in concretes. Due to its small particle size, however, MK can agglomerate, resulting in a nonuniform matrix and underperformance of the produced concrete. To address this issue, this paper exploits a low-cost, bio-based admixture—black tea extract (BTE)—to replace the traditional petroleum-based chemical admixture to enhance the dispersion and workability of MK blended cement mortars. Major biomolecules in the BTE such as caffeine, catechin, theanine, and theaflavin are rich in polyphenol, hydroxyl, and carboxylic acid groups, which can interact with cement particles and have profound effects on the hydration process and microstructure of the hydration products. Experimental studies showed that BTE does improve the workability of the MK blended cement mortar. More importantly, the BTE introduces significant change on the microstructure of the hardened pastes. Both the pores with size less than 50 nm and the total porosity of the hardened paste were significantly reduced, leading to a significant improvement in the micro- and macro-mechanical properties of the hardened paste. Experimental results suggest that up to 35% greater improvement in the compressive strength at 28 days was achieved using the proposed bio-admixture. Economic and environmental advantages of using the BTE as a renewable admixture were also illustrated through analyzing the cost–benefit, embodied carbon, and eco-efficiency of the MK blended mortars.

## 1. Introduction

The incorporation of supplementary cementitious materials (SCMs) into ordinary Portland cement (OPC) can improve the mechanical properties and reduce the clinker consumption required in concrete. Thus, the eco-efficiency of OPC-based concretes is improved, leading to a reduction in the carbon footprint and energy consumption [1]. Metakaolin (MK) is an aluminosilicate material produced by the calcination of kaolin clay, which has high pozzolanic reactivity [2,3] due to its high fineness. Replacing partial OPC with MK can refine the pore structure, improve the mechanical properties, and enhance the durability of concretes [2,3,4,5], all of which can be attributed to MK’s pozzolanic reactivity, as well as filler and nucleation effects [6,7]. 

The great potential of MK has been confirmed by extensive studies [2,3,4,5,6,7,8,9,10]. Due to their small particle size, however, MK grains conglomerate easily via strong van der Waals forces, resulting in a nonuniform MK blended concrete [11]. A previous study reported that the agglomeration of MK might reduce its pozzolanic reactivity, causing a lower compressive strength and poor microstructure [12]. In addition, the use of MK promotes a decrease in workability [13]. This suggests that the dispersion of the MK needs to be improved to achieve better efficiency and performance. To address this drawback, superplasticizer is used for adsorption on the particle surface, achieving deflocculation and dispersion of mineral grains via electrostatic repulsion forces and stereo effects [13,14,15]. 

Petroleum-based chemical admixtures are predominately used in the modern concrete industry. Manufacturing of these admixtures can consume a large amount of energy and generate high carbon emissions, as well as byproduct sludge. Since they are readily soluble in water, chemical admixtures can leach out of concretes, posing a potential threat to the environment. Thus, low-cost, clean, and environmentally friendly admixtures are needed, which can be used to replace the existing admixture to promote the dispersion of the MK in concretes. According to the data from International Tea Committee, the yearly world consumption of tea reached above 5.8 million tons in 2019 [16]. Furthermore, the amount of tea waste from factories accounts for 3–5% of the total production and can even reach 17–18% owing to improper harvesting procedures [17]. Black tea waste is a cheap source of biomass, which can be used as fertilizer, activated carbon, metal-based nanoparticles, biochar, and even construction materials [17,18]. The usage of black tea waste as construction material mainly involves “green corrosion inhibitors” and fiber materials [17]. However, there is very limited research on the use of black tea waste as a dispersing admixture of concrete to date.

**Figure 1:**
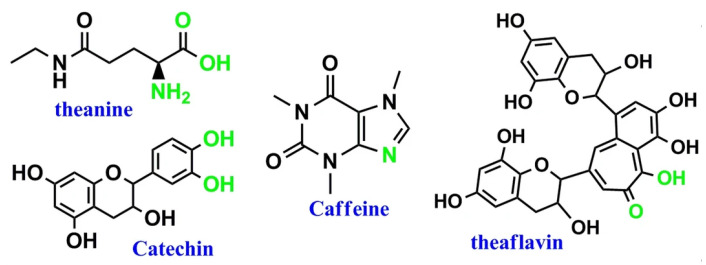
Four major biomolecules of black tea extract [21].

This study identified a natural product, black tea extract (BTE), as such an admixture for concrete, which was used in MK blended cement mortars to prevent the agglomeration of fine MK particles. BTE can be simply produced by soaking tea leaves or waste in boiling water to extract functional molecules from the boiling water. A previous study showed that caffeine, catechin, theanine, and theaflavin are the main biomolecules released from tea leaves into the BTE [19,20], as shown in Figure 1. These biomolecules are rich in polyphenol, hydroxyl, and carboxylic acid groups [21]. The polyphenol groups possess the capability to strongly bind to various surfaces through multiple interactions [22]. Thus, they can be easily adsorbed on the surface of minerals, especially for MK grains (~1–2 μm) due to their large surface area. Then, steric repulsion from the hydrophobic interactions (π–π stacking) of polyphenols prevents the aggregation or agglomeration of mineral particles [23,24]. More importantly, previous studies showed that polyphenols can capture calcium ions through a chelation reaction [25,26,27]. The calcium chelate complex facilitates a local super-saturation of Ca^2+^ [25] and inhibits the growth of minerals in the induction period, which provides more nucleation sites for the growth of minerals in the acceleration period [26]. As a result, a significant strength improvement has been achieved using a small dose of plant-based polyphenols as a strength enhancer [27]. Therefore, this study hypothesizes that plant extracts such as BTE rich in polyphenols can have similar functions to pure plant polyphenols in interacting with the hydration of OPC, eventually significantly enhancing the performance of the produced concrete. If this hypothesis is valid, plant extracts can be used to replace pure plant-based polyphenols in concrete as an eco-friendly admixture for concrete. Compared with pure polyphenols, polyphenol-rich plant extracts can be produced in a much cheaper and eco-friendlier way since the costly and environmentally harmful purification process to produce pure plant-based polyphenols is eliminated. Compared with existing petroleum-based admixtures, BTE is potable, posing no threat to the environment. 

This study was designed to test this hypothesis. To this end, an experimental program was carried out to evaluate the effect of using the BTE as an admixture on the performance of MK blended cement mortars. Hydration kinetics, X-ray powder diffraction analysis (XRD), thermal gravimetric analysis (TGA/DTG), mercury intrusion porosimetry (MIP), and nanomechanical property analysis were also carried out to delve into the mechanisms of the observed improvement in the performance of the mortar. In addition, an environmental impact assessment was conducted to evaluate the eco-efficiency of the BTE.

## 2. Materials and Methods

### 2.1. Materials

Type I Portland cement was used; its chemical composition is presented in Table 1. The high-reactivity metakaolin was obtained from Powerpozz with the chemical composition shown in Table 1. This type of metakaolin has a D_50_ particle size of 3 μm, 99.9% of particles finer than 16 μm. Natural river sand with a fineness modulus of 2.82 was used as fine aggregate, purchased from Red Eagle Sand & Gravel in Alabama. These fine aggregates were oven-dried for 12 h at 110 °C and then cooled for 12 h before being mixed with other ingredients. Rosekandy premium black crush–tear–curl (CTC) granule tea with rich antioxidants was obtained from Rosekandy Tea Estate, India, containing 0.6% total soluble solids.

### 2.2. Preparation of Black Tea Extract

Three different black tea dosages were used in this study: 0.5%, 1%, and 2% by weight of total mixing water in the mix proportions. Black tea was weighed in reusable coffee cup filters and added to 400 g of boiling water. After brewing for 1 h at 100 °C, the tea solution was vacuum-filtered through Whatman No. 44 filter paper. The obtained black tea extract was cooled to ambient temperature before being used in a mix. To compensate for the evaporated water, some DI water was added to the cooled BTE to reach the required amount of mixing water shown in Table 2.

### 2.3. Mix Design and Preparation of Mortars

Table 2 presents the mix proportion of produced mortar samples with a water-to-binder ratio (*w*/*b*) of 0.55. The control group was named T0M0 and produced with only cement, water, and sand. The experimental groups were named TxMy, where Tx indicates the BTE obtained from boiling the black tea with an amount of x% by the weight of the mixing water, while My indicates the cement replaced by MK at y% of the weight of total cementitious materials. Four dosages of the black tea were adopted to produce the BTE: 0, 0.5%, 1%, and 2% by weight of the cement. Four contents of the MK were used to replace cement: 0%, 5%, 10%, and 15% by weight of the cement. All mortar samples were cast into 50 mm × 100 mm cylinders and then moist-cured at ambient temperature.

### 2.4. Testing Methods

#### 2.4.1. Flowability Test

According to ASTM C1437-15 [28], the flowability of the fresh mortar samples could be measured using a flow table to evaluate whether BTE can increase the workability of the mortars. 

#### 2.4.2. Isothermal Calorimetry Test

This test was carried out to examine the effect of BTE on the hydration of cement up to 72 h according to ASTM C1679-17 [29], which was conducted using the Calmetrix I-Cal 4000 isothermal calorimeter at the ambient temperature of 23 °C. Test samples were prepared by mixing 50 g of cementitious materials, including cement and metakaolin (MK), with 25 g of DI water or BTE. The actual amounts of cement and metakaolin were calculated on the basis of the replacement percentage in each group. The measured thermal heat and thermal power were normalized by the total weight of the total cementitious material. 

#### 2.4.3. Compressive Strength of the Produced Mortars

The compressive strength of 50 mm × 100 mm cylindrical mortar samples was measured using a Humboldt compressive strength testing machine. Three duplicated samples were tested at 3 days, 7 days, and 28 days in compliance with ASTM C39-18 [30] with a constant loading rate of 0.25 MPa/s. During testing, capping pads were used to level the cylinders, eliminating the error from unevenness.

#### 2.4.4. XRD and TGA

XRD and TGA/DTG were used to investigate the influence of BTE on the hydration products of cement. To this end, the paste samples cured in sealed conditions for 7 days and 28 days were pulverized and ground into fine powders. The ground powders were then sieved through a #100 mesh (150 µm) for XRD or TGA measurements. The TGA measurement was conducted using a PerkinElmer STA 8000 instrument. The sample was stabilized at 30 °C for 30 min, followed by heating from 30 °C to 850 °C with a rate of 10.00 °C/min. 

#### 2.4.5. MIP

A Micromeritics AutoPore IV 9500 was used for the MIP analysis. The testing samples used in this test were obtained by crushing the produced cement pastes after 28 days of curing in sealed conditions. 

#### 2.4.6. Nanoindentation

For nanoindentation testing, the paste samples were prepared under sealed curing for 28 days. The sample pieces were cast with epoxy resin, and then polished with SiC sanding paper and felt cloths. A Histron TI950 nanoindenter was used to generate an 11 × 11 grid with a 10 μm spacing using the partial unloading method as described in [26]. The maximum load was chosen as 1 mN. In order to eliminate the creep, surface roughness, and size effects, partial loading and unloading modes were chosen to achieve each indent [31]. The elastic modulus at each indent was calculated using the Oliver and Pharr method [32]. 

For heterogeneous and multiphase materials such as cement paste, the coupling data of elastic modulus can be processed using the Gaussian mixture model (GMM) with an expectation–maximization algorithm (EM) [24,25]. An elastic modulus smaller than the C–S–H particle stiffness (E ≤ 60 GPa) was used to identify C–S–H phases [33]. On the basis of the deconvolution results, four phases of hydration products (excluding anhydrous clinker minerals) could be identified: loosely packed C–S–H or porous phases (PP), low-density C–S–H (LD), high-density C–S–H (HD), and ultrahigh-density C–S–H (UHD, a mix of C–S–H and portlandite) [9,34,35].

## 3. Results and Discussion

### 3.1. Effects of Black Tea Extract on the Performance of Mortar

#### 3.1.1. Flowability

**Figure 2:**
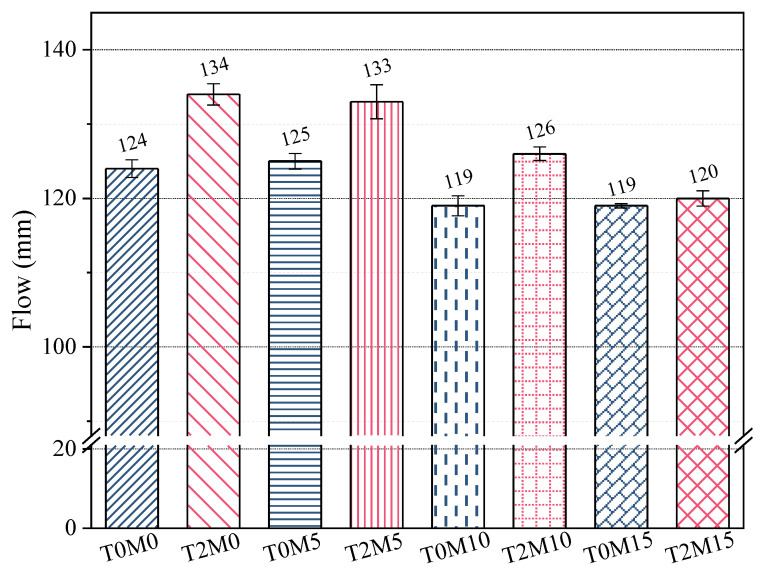
Flowability of MK blended mortars mixed with black tea extract.

The results of workability of fresh mortars are presented in Figure 2. With the increasing replacement of MK, the workability of mortars was reduced in comparison with the control group produced without MK. This is expected since highly active metakaolin has a smaller size, larger surface area, and higher water absorption than cement. When mixing mortars with the BTE, the workability was increased by 10 mm, 8 mm, 7 mm, and 1 mm for the mortar samples with MK in dosages of 0%, 5%, 10%, and 15%, respectively. This indicates that the BTE could serve as a water reducer for the MK blended mortars. The biomolecules in the BTE could be easily adsorbed onto the surface of cement or metakaolin particles. The phenolic groups or hydroxyl groups adsorbed onto the surface of minerals increased the steric repulsion between individual minerals, including cement and MK [36], thus dispersing these particles easily. Very little improvement was achieved by T2M15 compared to T0M15, suggesting that, for such a high content of MK, 2% black tea was not sufficient to produce a satisfactory water-reducing effect. In addition, the interaction between the BTE and MK could contribute to a loss of workability, as discussed in later sections.

#### 3.1.2. Hydration Kinetics

**Figure 3:**
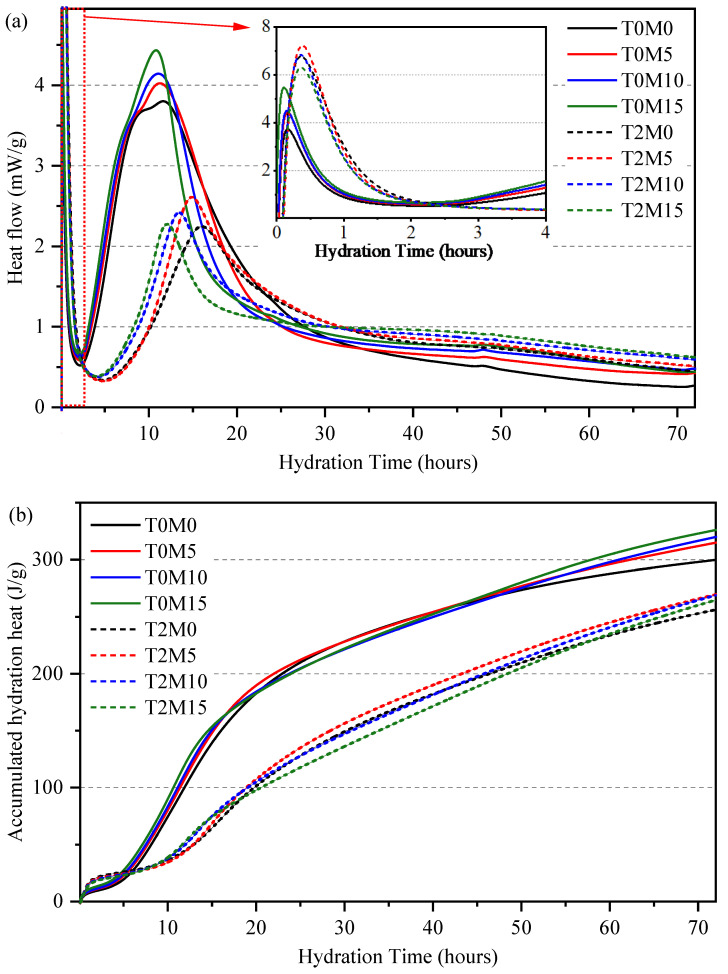
Hydration kinetics of the metakaolin blended cement pastes with black tea extracts: (**a**) thermal power; (**b**) accumulated heat.

To evaluate the effect of the BTE on the hydration of the MK blended cement, the hydration kinetics was investigated using isothermal calorimetry, and the results are presented in Figure 3. This figure shows that MK could accelerate the hydration of the cement as indicated by the increased heat flow rate at the main peak and the accumulated hydration heat in the samples without the BTE. This is because the high reactivity and large surface area of MK promote the nucleation rate during the hydration of cement. In addition, the addition of MK increased the aluminate/silicate ratio in the blended system, interfering with the hydration of the aluminate phases. In the plain Portland cement paste sample (T0M0), there was a small and broad shoulder peak generated by the hydration of the aluminate phases. This peak became higher and earlier with the increase in replacement level of MK in the blended system, consistent with a previous study [8]. 

The addition of BTE significantly retarded the hydration of cement, as evidenced by the delayed onset of the acceleration, lower heat released rate at the main hydration peak, rightward shift of the heat flow curves, and lower accumulated hydration heat until 72 h. In the presence of BTE, increasing the amount of MK led to earlier onset of the acceleration period, a higher slope of the acceleration period, and a leftward shift of the heat flow curves, suggesting that the hydration of cement paste with BTE could be accelerated by using MK in the paste. This is because biomolecules in the BTE were more easily adsorbed onto the surface of the MK particles due to their larger surface area. As a result, fewer biomolecules are adsorbed onto OPC particles when more MK was used, reducing the retarding effect of the BTE on the hydration of the cement.

In addition, there was a rapid heat evolution occurring at the very beginning of the hydration (initial period), caused by the wetting of the cement, dissolution of ions, and hydration of aluminate phases [37]. In the presence of MK only, this first aluminate peak increased in intensity, indicating that more ettringite was produced. However, the BTE significantly enhanced the heat flow rate in this period and delayed the initial period by around 1 h. It is not yet clear how the BTE contributes to this peak. One possible reason is that the biomolecules in the BTE react with the metal ions via the chelate reaction or participate in the conversion of gypsum hemihydrate into ettringite.

#### 3.1.3. Compressive Strength

**Figure 4:**
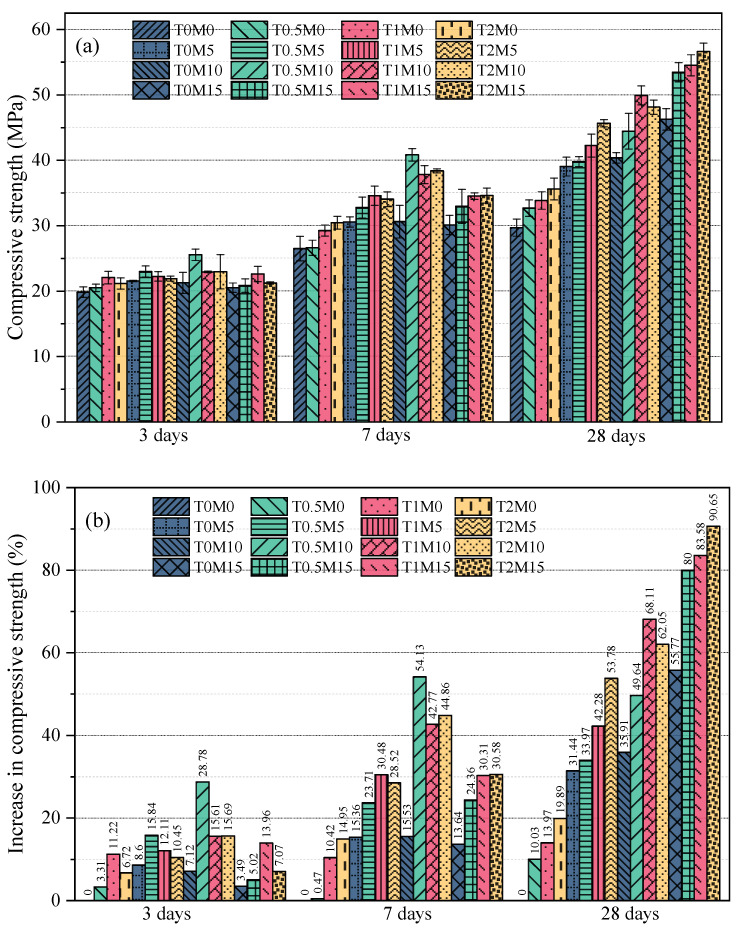
The compressive strength improvement by black tea extract and metakaolin: (**a**) the compressive strength; (**b**) the increasing percentage.

Compressive strengths are given in Figure 4a, and their relative increase percentages with respect to the control group (T0M0) are shown in Figure 4b. It can be seen that the compressive strengths of mortars at all ages were significantly enhanced when using MK to partially replace Portland cement. This is because the MK blended mortars had denser microstructures induced by the filler effect of MK and the pozzolanic reaction between MK and portlandite. In addition, the hydration of the cement was also accelerated by the seeding effect provided by MK. Similarly, the compressive strengths of the mortar samples using the BTE surpassed those of the control at all ages. This improvement was attributed to the improved workability of mortars induced by the uniform dispersion of the cement particles via steric repulsion. At 3 days, the maximum strength improvement was achieved by T0.5M0, and a higher concentration of the BTE could reduce the strength at this age because of the retarding effect of the BTE. At 7 days and 28 days, the strength improvement of the mortars increased with the concentration of the BTE, suggesting that the retarding effect of the BTE diminished at the late ages of the mortar. As a result, a 20% strength improvement at 28 days was reached by T2M0. 

The benefit of adding the BTE to the MK blended cement mortars was clearly demonstrated by the improvement in the compressive strengths of these mortars. All MK blended mortars with the BTE reached higher strength than those without the BTE at all ages. At 3 days, maximum strength improvements for the mortars with 5% MK and 10% MK were achieved when using 0.5% black tea at 15.84% and 28.78%, respectively. When the content of black tea was increased to 1% and 2%, the compressive strengths of these mortars were slightly reduced. This is because a stronger retarding effect was induced by using more tea to make the BTE. When 15% MK was used in the mortar, the maximum strength improvement at this age was achieved when using 1% black tea (T1M15). Since more MK was used in this mortar, the greater retarding effect of the BTE was offset by the accelerating effect of MK. As a result, the maximum strength improvement was reached when using 1% black tea. A similar effect of the content of the black tea on the strength of the produced mortars could be observed at 7 days. After 28 days of curing, the retarding effect of the BTE diminished, and a positive correlation existed between the content of black tea and the compressive strength improvement of the mortar. The great potential of using the BTE to promote the application of MK was clearly revealed by the 90.65% improvement in compressive strength at 28 days achieved by T2M15. 

#### 3.1.4. Hydration Products (XRD and TGA)

**Figure 5:**
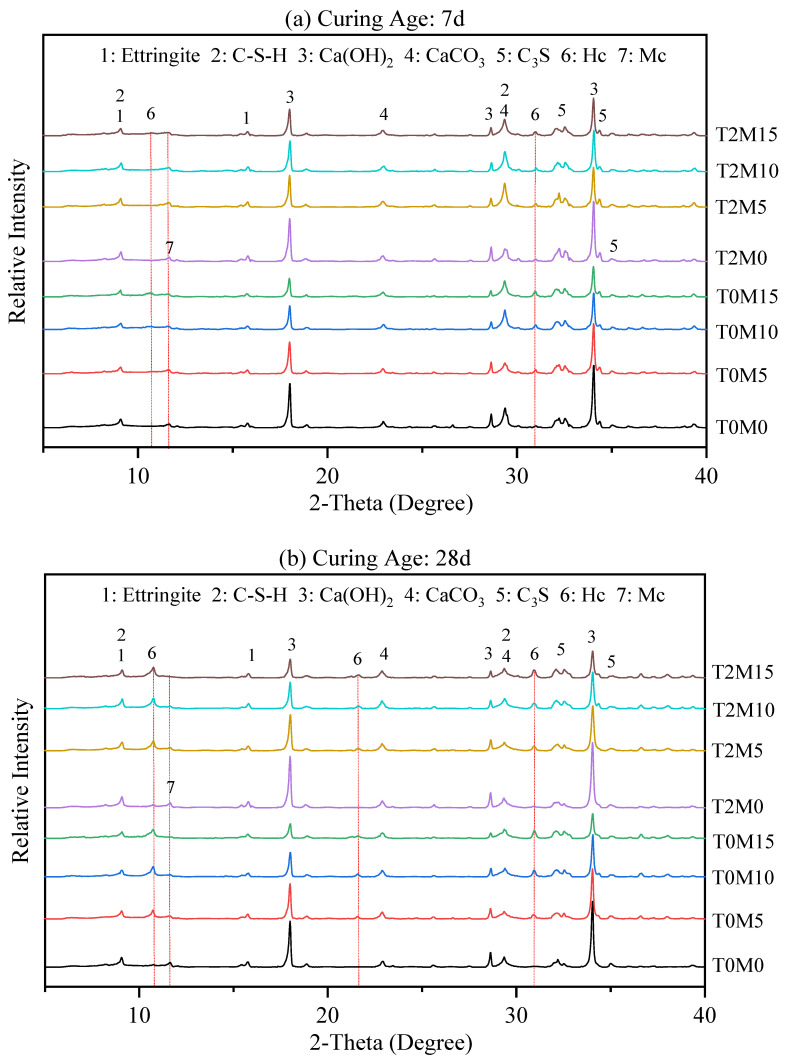
XRD patterns of MK blended cement pastes with the BTE at (**a**) 7 days and (**b**) 28 days. At later ages, the formation of hemicarboaluminate (Hc) was observed in MK blended system, while monocarboaluminate (Mc) was mainly observed in pure cement mortars.

Figure 5 presents the XRD pattern of the produced pastes with BTE at 7 days and 28 days. Typical crystalline hydration products such as C–S–H, CH, ettringite, calcite, and alite from non-hydrated cement particles could be identified in the results. Furthermore, the MK blended system showed the formation of hemicarboaluminate (Hc) and a small amount of monocarboaluminate (Mc) after 7 days, whereas there was only Mc in the pure cement system. This is because MK could react with calcium carbonate and portlandite to form Hc, AFm, and C–A–S–H gel [8]. It should be mentioned that the CH content in a Portland cement paste represents the hydration degree of the cement. After MK is added, less CH is produced by the hydration of cement because of the dilution effect and the consumption of some of the produced CH via the pozzolanic reaction between CH and MK. As a result, the CH content in the MK blended cement pastes was reduced with the increase in content of MK, resulting significantly lower than that in the pure cement paste.

When the BTE was used to prepare the MK blended cement pastes, the contents of CH in the produced pastes were significantly higher than in those without using the BTE at 7 days. This indicates that the BTE could facilitate the hydration of the cement to produce more CH, suppress the pozzolanic reaction of MK to consume less CH at this age, or both [3]. It can be found that more CH was present in T2M0 than in T0M0 (pure cement paste), suggesting that adding the BTE produced more CH in the pure cement mortar at 7 days. Figure 5a also shows that the CH contents in the three MK blended cement pastes with BET (T2M5, T2M10, and T2M15) were almost identical, regardless of the content of MK. This suggests that the pozzolanic reaction between CH and MK was very limited at this age. As a result, very little CH was consumed, leading to a similar CH content in these pastes. Clearly, the presence of the BTE drastically slowed the pozzolanic reaction between CH and MK due to the adsorption of biomolecules in the BTE onto the surface of MK particles. This blocking effect of the BTE on the pozzolanic reaction seemed to disappear at 28 days. At this age, the content of CH in these pastes with the BTE decreased with the content of MK. Clearly, more CH was consumed in the paste with more MK.

**Figure 6:**
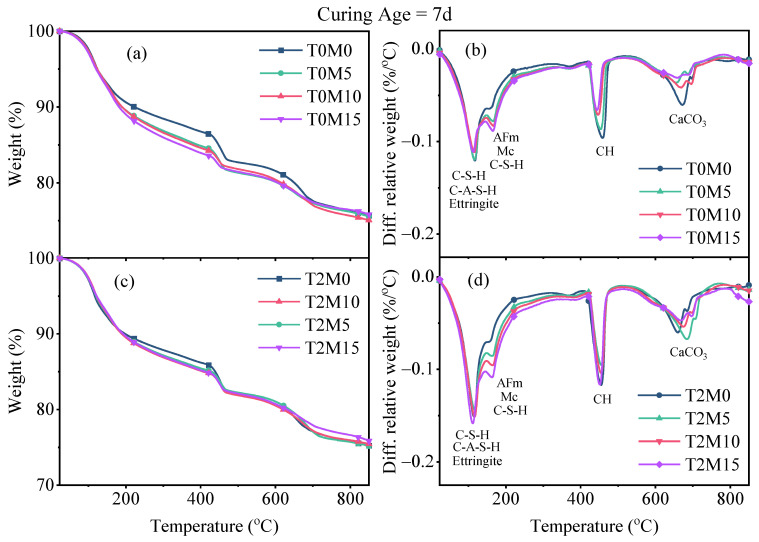
TGA curves of the produced MK blended pastes with BTE after 7 days of sealed curing: (**a**) TGA curves of pastes made with MK; (**b**) DTG curves of pastes made with MK; (**c**) TGA curves of pastes made with MK and BTE; (**d**) DTG curves of pastes made with MK and BTE.

**Figure 7:**
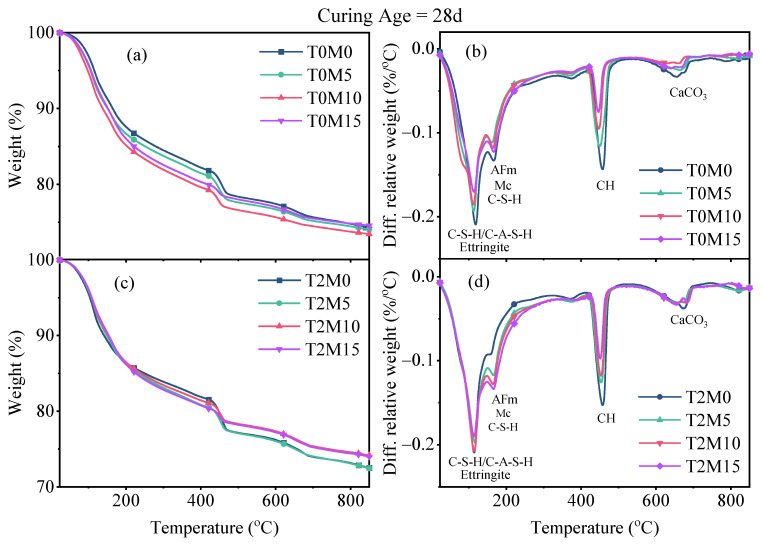
TGA results of the produced MK blended pastes with BTE after 28 days of sealed curing: (**a**) TGA curves of pastes made with MK; (**b**) DTG curves of pastes made with MK; (**c**) TGA curves of pastes made with MK and BTE; (**d**) DTG curves of pastes made with MK and BTE.

TGA was conducted to further identify the possible reasons behind the effect of the BTE on the compressive strength of MK blended cement mortars, and the results are illustrated in Figure 6 and Figure 7. The decomposition of specific phases of the hydration products can be obtained on the basis of the weight loss according to the peak intervals shown in DTG curves [38]. The first weight loss peak at around 100 °C came from the decomposition of ettringite and the dehydroxylation of C–S–H and C–A–S–H phases. The C–S–H phase exhibited weight loss from 40 °C to 600 °C due to the dehydroxylation or loss of interlayer water. There was a subsequent sharp peak between 400 °C and 500 °C in the DTG curves, related to the evaporation of water in CH. Peaks above 550 °C were due to the decomposition of carbonates in calcite.

According to the DTG results, the mass loss between 40 °C and 550 °C was regarded as the amount of bound water using Equation (1). The weight loss related to the decomposition of CH can be measured by integrating the peak area between 400 °C and 500 °C in DTG curves [36]. This tangential method can correct the overestimation of CH and exclude the weight loss caused by the ongoing decomposition of C–S–H in this temperature interval [38]. Then, the amount of CH can be calculated on the basis of the molecular masses of CH (74 g/mol) and water (18 g/mol), as given by Equation (2). The quantifications of the bound water and CH were normalized to the dry weight of binders at 550 °C [39].

(1) $$W_{\left[H_{2}O\right]}=\frac{M_{40}-M_{550}}{M_{550}}\times 100(\%),$$

(2) $$W_{\left[Ca{\left(OH\right)}_{2}\right]}=\frac{M_{400}-M_{500}}{M_{550}}\times \frac{74}{18}\times 100(\%),$$

where M_40_, M_400_, M_500_, and M_550_ are the dry weights of the sample at 40 °C, 400 °C, 500 °C, and 550 °C, respectively.

**Figure 8:**
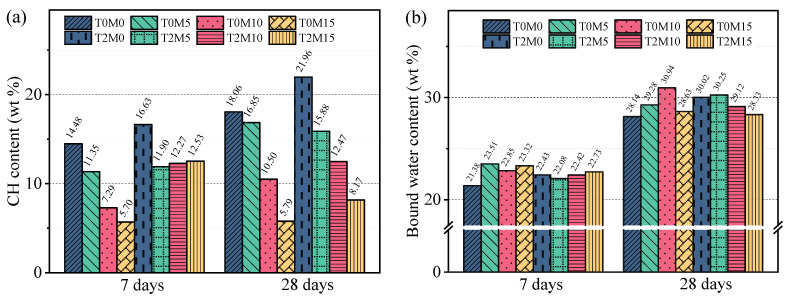
Mass loss of different hydration products and total bound water of MK blended pastes with BTE obtained from the TGA results: (**a**) CH; (**b**) bound water.

The amounts of bound water and CH determined from the TGA data are presented in Figure 8. In the absence of BTE, more MK in the blended system led to less CH at 7 days and 28 days. As mentioned earlier, this reduction in CH was induced by the pozzolanic reaction of MK, as well as the dilution effect of the blended system. The addition of BTE increased the amount of CH even in the MK blended system, indicating that the BTE could promote the hydration degree of the cement. At 7 days, all MK blended cement pastes with the BTE possessed a similar content of CH around 12%, regardless of the content of MK in the sample. This agrees well with the XRD results shown in Figure 5 and can be attributed to the absorption of the BTE onto the MK surface, suppressing its pozzolanic reactivity at the early age. This suppressing effect on the pozzolanic reaction of the BTE gradually disappeared as the curing time increased to 28 days. At this age, the CH content decreased with the increase in MK content because more CH was consumed by the pozzolanic reaction. 

The addition of MK increased the total bound water of the hydration products. In particular, the bound water at 28 days increased with the content of MK except for the 15% replacement. In the MK blended system, the amount of bound water depended on the hydration degree of the cement and the pozzolanic reaction degree of the MK. With a low replacement level, the degree of pozzolanic reaction of MK was higher since more CH was available for the pozzolan to react with [3]. For this reason, the amount of bound water was reduced in the blended sample with 15% MK, as shown in Figure 8b. The presence of the BTE increased the bound water of the pure Portland cement paste (pure cement) at 7 days and 28 days, indicating that the hydration degree of the cement was improved by the BTE. The addition of the BTE to the MK blended system altered the relationship between the bound water and the MK replacement level. At 7 days, there was a similar amount of bound water for all three replacement levels of MK (T2M5, T2M10, and T2M15). At 28 days, the amount of bound water decreased gradually with the increase in MK replacement level. One possible reason is that the degree of pozzolanic reaction was lower for a higher content of MK [3]. The detailed mechanism related to the change of bound water in this binary blended system is still unclear and will be investigated in future research.

### 3.2. Pore Structure Analysis

**Figure 9:**
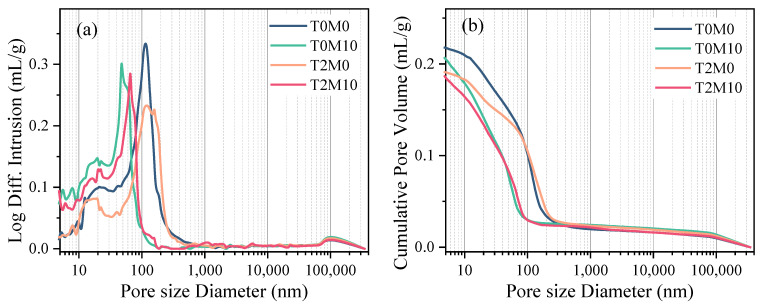
Pore size distribution of the MK blended pastes with BTE: (**a**) log differential intrusion; (**b**) cumulative intrusion.

The pore structures of the blended cement pastes after 28 days of curing are illustrated in Figure 9, and the results of total porosity are summarized in Table 3. As shown in Figure 9a, the pore structure was significantly refined by the addition of MK, leading to a drastic reduction in pores with sizes of 50–1000 nm and a clear shift toward finer pores. These improvements in pore structure could be attributed to the nucleation effect, pozzolanic effect, and filler effect of MK [3].

The addition of the BTE could also significantly change the pore structures of the pastes, but in a different way. Similar to adding MK, the overall porosity of the pastes was reduced by the BTE. However, this reduction was not achieved through refining the pore sizes. As shown in Figure 9a, the peak of the capillary pore size distribution curve was shifted to the right upon adding the BTE, suggesting that these pores were enlarged. However, the heights of the peaks of the samples with the BTE (T2M0 and T2M10) were lower than those without the BTE, suggesting that the total number of pores was reduced by the BTE. More importantly, most capillary pores with a size smaller than 50 nm were gel pores and inter-hydrate pores. This suggests that the packing density of C–S–H was significantly increased by the addition of the BTE. This densifying effect of the BTE could be attributed to the biomolecules in the BTE. These biomolecules are rich in catechol groups, which have strong adhesion and multiple interactions with calcium ions. The obtained calcium chelate can offer binding sites for the nucleation of C–S–H and even compact the loose hydration products into denser grains in nanoscale. Such a drastic change in the microstructure of the samples inevitably affected the compressive strength of the mortar samples. As a result, much higher compressive strength was achieved by these MK blended cement mortars mixed with BTE, as observed in Figure 4.

### 3.3. Grid of Nanoindentation

**Figure 10:**
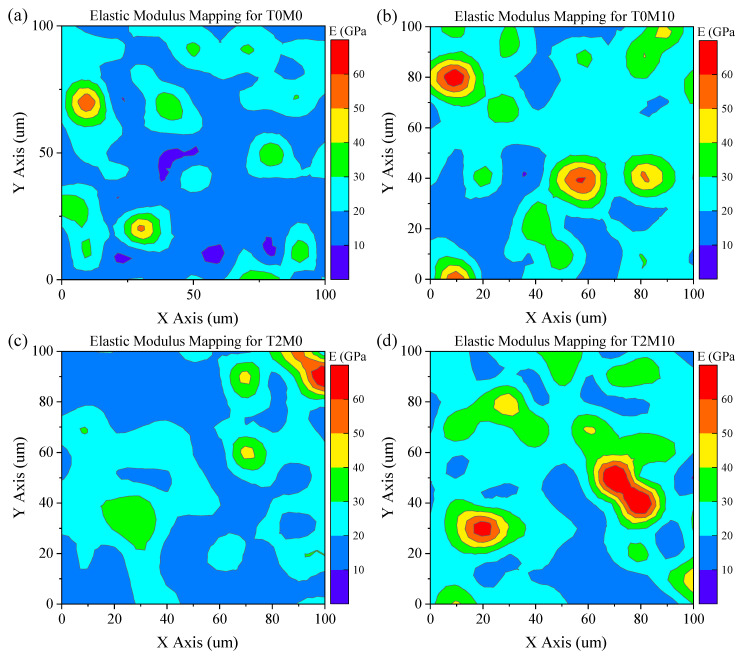
Contour mapping of elastic modulus obtained from grid of nanoindentation: (**a**) T0M0; (**b**) T0M10; (**c**) T2M0; (**d**) T2M10.

Nanoindentation was carried out to examine the effect of the microstructure change induced by the BTE and MK on the mechanical properties of the hydration products. The elastic modulus mappings in the corresponding indented area are shown in Figure 10 for four pastes. The refining effect of MK on the elastic modulus of the hydration products was revealed by comparing the mappings of the pure cement paste (Figure 10a) to that with 10% MK (Figure 10b). The presence of the MK significantly improved the elastic modulus of the blended pastes compared to the control group, especially for the region with a modulus within 20–30 GPa. Similarly, the elastic modulus of the pure cement paste was also improved by the addition of the BTE, as shown in Figure 10c. A highest elastic modulus is achieved by T2M10 due to the synergistic effect of MK and BTE, as also revealed by the MIP analysis shown in Figure 9.

The hydration product C–S–H is normally made of the same compacted (11% porosity) and nanosized building blocks but with different aggregation density [40]. The packing density of C–S–H is positively related to the elastic modulus of the hydration products [41,42]. Therefore, the enhanced modulus of elasticity induced by adding the BTE indicates that the microstructure or the packing density of the hydration products was improved by the addition of the BTE, which agrees well with the MIP results shown in Figure 9. This change in microstructure and micromechanical properties led to a higher compressive strength of the mortars, as shown in Figure 4.

**Figure 11:**
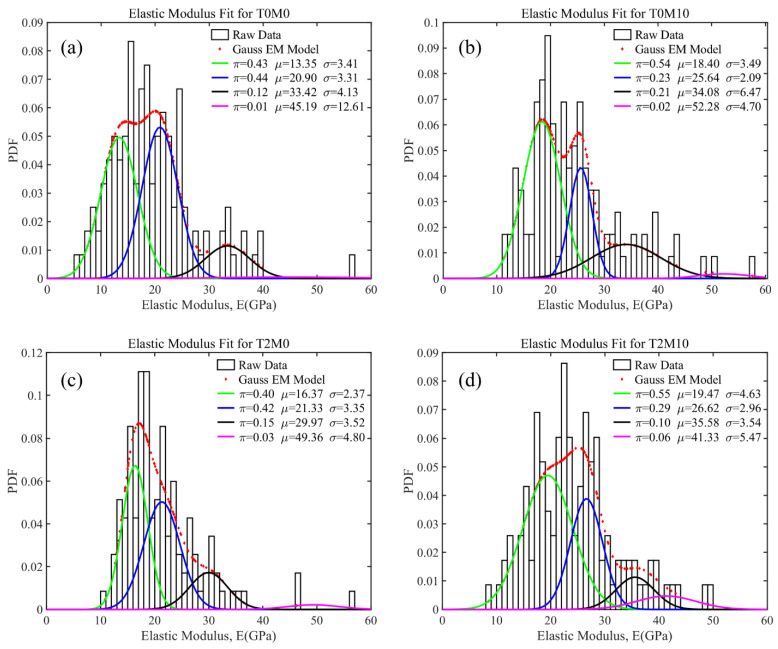
Deconvolution results of elastic modulus for the MK blended pastes with BTE: (**a**) T0M0; (**b**) T0M10; (**c**) T2M0; (**d**) T2M10.

For cement pastes, four phases of hydration products could be identified on the basis of the deconvolution results in ascending order of elastic modulus. The deconvolution results of elastic modulus are shown in Figure 11 and summarized in Table 4. The addition of MK could significantly improve the elastic modulus of each hydration product, especially for the porous phases, which was improved from 13.35 GPa in the control group (T0M0) to 18.40 GPa in the sample with 10% MK (T0M10). Comparing the volume fractions of each phase in T0M0 (Figure 11a) and T0M10 (Figure 11b), adding MK to the paste led to an increase in the porous phase and HD C–S–H, as well as a decrease in LD C–S–H. This is because MK generates more C–A–S–H than C–S–H in the blended system, corresponding to the porous phases during the deconvolution process. Due to the nucleation filler effects of MK, capillary pores in the hydration products were reduced, as revealed in Figure 9, leading to a 23.92% increase in the average elastic modulus of C–S–H compared to that of the control group. 

The addition of the BTE slightly changed the volume fraction of each phase of C–S–H compared with the control group, as shown in Figure 11c. However, the elastic moduli of the dominant phases including porous phases and LD C–S–H were significantly increased by the BTE. As a result, the average elastic modulus of all C–S–H phases was increased by 10.72%. This suggests that the addition of BTE could improve the packing density of the hydration products, consistent with the pore size distribution shown in Figure 9. The improvement in hydration products could also generate a higher compressive strength of mortars, as revealed in Figure 4.

The necessity of adding the BTE to the MK blended cement mortar was revealed by the highest elastic modulus reached for each phase of the hydration products expect UHD C–S–H and the highest overall average elastic modulus of all phases, as shown in Figure 11d. Specifically, less HD C–S–H, more LD C–S–H and UHD C–S–H were produced in the MK blended pastes with the BTE than in those without BTE. This agrees with the MIP result shown in Figure 9, which suggests that adding the BTE reduced the number of pores with a size smaller than 50 nm but increased the number of pores with a size between 50 nm and 100 nm. As a result, more UHD C–S–H and LD C–S–H and less HD C–S–H were produced in the MK blended paste with BTE.

### 3.4. Environmental Impact Assessment and Cost–Benefit Analysis

The major advantage of using a natural product such as BTE to replace existing nonrenewable chemical admixtures for concrete is its environmental and economic benefits, as presented in Table 5. The material costs in Table 5 were determined on the basis of local quotation prices. It should be noted that the carbon footprint of black tea itself is around 12 metric kg eq. CO_2_/kg due to the CTC process [43]. However, the extract can be made from unprocessed tea leaves or by recycling used tea leaves, adding a negligible carbon footprint to the concrete and eliminating the negative environmental impact associated with the disposal of waste materials. More importantly, many other plants rich in polyphenols can be used to replace black tea to make extracts which may have a similar function to the BTE. The transportation of construction materials is the main contributor to total carbon emissions and cost. The embodied carbon and cost of BTE can be estimated on the basis of key information related to transportation found in the literature: 0.0075 kgCO_2_e/kg/100 km [44] and 0.039 USD/ton/100 km [45]. Since the reusage of BTE as a construction material can be localized, the distance used in this study was assumed as 200 km. Thus, the embodied carbon and cost of BTE were obtained as 0.015 kgCO_2_e/kg and 0.078 USD/ton. 

Another important benefit of using BTE in the MK blended system is that the eco-strength efficiency of the produced concrete could be significantly improved. The eco-strength efficiency can be regarded as the inverse of the CO_2_ intensity indicator, which is defined as the amount of CO_2_ emitted to deliver one unit of performance [50].

(3) $$Eco-efficiency=\frac{Compressive\;strength\;at\;28d\;}{Embodied\;carbon}.$$

In addition, the cost–benefit is also a vital factor for blended concrete in the construction industry. The cost–benefit can be defined as the level of performance, i.e., compressive strength at 28 days, for one unit of cost (per dollars).

(4) $$Cost-benefit=\frac{Compressive\;strength\;at\;28d\;}{Total\;material\;prices}.$$

**Figure 12:**
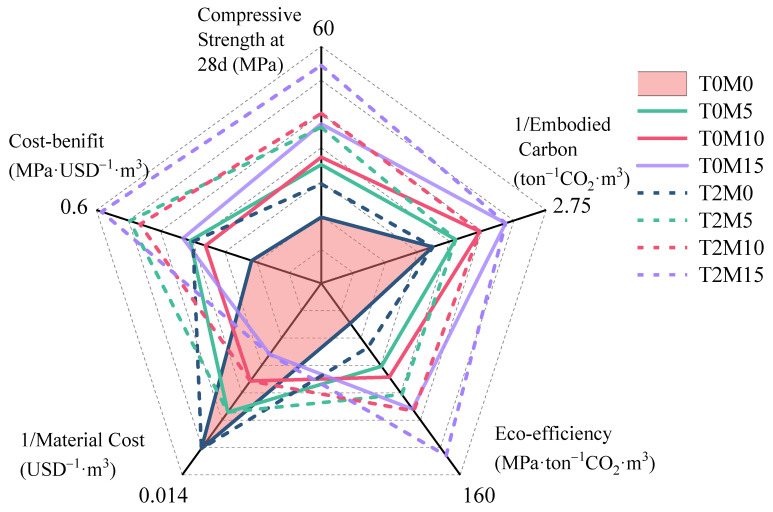
Five-dimensional assessment considering mechanical properties, embodied carbon, eco-efficiency, material cost, and the cost–benefit (strength per unit cost) of blended mortars. The calculated embodied carbon and material cost were based on 1 m^3^ of mortars.

According to the mix proportions of the mortars, the embodied carbon, the eco-efficiency, the material costs, and the cost–benefit of the MK blended mortars with BTE were estimated, and the results are summarized in Figure 12. It can be concluded that a higher substitution of OPC with MK led to lower total carbon emissions of the mixture. Since the cost of MK is relatively high compared with OPC, the price of the mixtures increased with the replacement level. However, due to the significant strength enhancement achieved by MK, the eco-efficiency and cost–benefit increased gradually with the content of MK. This indicates that MK blended concrete has better sustainability and a higher cost efficiency.

A significant jump in eco-efficiency and cost-efficiency was reached upon adding the BTE, as shown in Figure 12. This can be attributed to the significant enhancement of strength due to the unique ability of the BTE to densify the hydration products. The addition of BTE could increase both the eco-strength and cost–benefit efficiency in the MK blended system. By comparing T0M15 and T2M10, 2% BTE could achieve a similar effect to replacing 5% cement with MK in terms of eco-efficiency and cost–benefit, demonstrating the great potential of BTE as a natural admixture for MK blended mortars. In addition, T2M15 had the best overall performance in terms of cumulative benefits, including good mechanical properties, a low carbon footprint, and high efficiency for sustainability.

## 4. Conclusions

This work examined the feasibility of using black tea extract to replace pure plant-based polyphenol as a renewable admixture to enhance the performance of MK blended mortars. The experimental studies carried out in this study clearly demonstrated the benefits of using of BTE in the MK blended system. A few major conclusions can be drawn from the experimental program.

(1) The addition of a small amount of BTE could remarkably improve the workability of blended mortar when the content of MK was no more than 10%, thanks to the steric repulsion caused by the polyphenol groups of the BTE. If the content of MK was higher than 15%, an excessive amount of BTE was required to achieve a significant improvement in the workability of the mortar. However, this may drastically reduce the early age strength of the mortar due to the significant retarding effect of the BTE on the hydration of the cement. For practical applications, the dose of BTE should be determined appropriately to avoid excessive loss of the early age strength.
(2) The presence of BTE increased the bound water and CH contents in the hardened pastes at later ages, indicating that the hydration of cement was promoted by the BTE, even though the hydration at the early age was retarded.
(3) A profound change in the microstructures of the produced pastes was induced by the addition of the BTE. In addition to the reduction in the total porosity of the pastes, the number of pores with a size smaller than 50 nm in both the OPC and the MK blended system was significantly reduced by the BTE. Although these small pores are usually regarded as almost harmless with respect to the strength of the concrete, this study showed that they had a profound effect on the micromechanical properties of the hardened paste. The average elastic modulus of the hydrated phases obtained through nanoindentation was drastically improved upon adding the BTE.
(4) A significant improvement in the mechanical properties of the hydration products was induced by the BTE, leading to a drastic improvement in the compressive strength of the produced mortar. For the cement mortar, up to 20% improvement in the compressive strength at 28 days could be achieved by the addition of BTE. For the MK blended system, over 90% improvement in the compressive strength of the mortar at 28 days was achieved.
(5) The environmental impact assessment and cost–benefit analysis revealed the potential of using a natural admixture such as BTE in MK blended mortars to achieve high eco-strength and cost–benefit efficiency, leading to a reduction in the carbon footprint and energy consumption of the produced concrete.

## Figures and Tables

**Table 1:** Chemical composition of Type I Portland cement and metakaolin (MK).

Composition	SiO_2_	Al_2_O_3_	CaO	Fe_2_O_3_	TiO_2_	MgO	Na_2_O	K_2_O	P_2_O_5_	LOI
Cement (%)	22.93	4.68	64.04	2.41	0.20	3.38	0.23	0.76	0.08	0.82
MK (%)	59.4	30.8	0.1	1.4	0.5	2.2	2.7	1.3	-	0.68

**Table 2:** Mix design of mortar samples.

Mix ID	Water (kg/m^3^)	Tea Solution (kg/m^3^)	Black Tea (wt.% of Water)	Cement (kg/m^3^)	MK (kg/m^3^)	Sand (kg/m^3^)
T0M0	264		-	480	-	1593
T0M5	264		-	456	24	1593
T0M10	264		-	432	48	1593
T0M15	264		-	408	72	1593
T0.5M0	-	264	0.5	480	-	1593
T0.5M5	-	264	0.5	456	24	1593
T0.5M10	-	264	0.5	432	48	1593
T0.5M15	-	264	0.5	408	72	1593
T1M0	-	264	1	480	-	1593
T1M5	-	264	1	456	24	1593
T1M10	-	264	1	432	48	1593
T1M15	-	264	1	408	72	1593
T2M0	-	264	2	480	-	1593
T2M5	-	264	2	456	24	1593
T2M10	-	264	2	432	48	1593
T2M15	-	264	2	408	72	1593

**Table 3:** Porosity of produced MK blended pastes with BTE.

Group	T0M0	T0M10	T2M0	T2M10
Porosity (%)	31.13	28.65	27.98	26.01

**Table 4:** Elastic modulus of different phases of C–S–H from grid of nanoindentation of MK blended cement pastes with BTE.

Specimen	Volume Fraction				Mean (GPa)				Avg. (GPa)
	PP	LD	HD	UHD	PP	LD	HD	UHD	
T0M0	0.43	0.44	0.12	0.01	13.35	20.90	33.42	45.19	19.40
T0M10	0.54	0.23	0.21	0.02	18.40	25.64	34.08	52.28	24.04
T2M0	0.40	0.42	0.15	0.03	16.37	21.33	29.97	49.36	21.48
T2M10	0.55	0.29	0.10	0.06	19.47	26.62	35.58	41.33	24.47

**Table 5:** Embodied carbon and material costs of raw materials.

Materials	Embodied Carbon (Metric ton Eq. CO_2_/Metric ton)	Material Costs (USD/Metric ton)
Type I Portland cement	0.82 [46]	124
Metakaolin	0.33 [47]	400
Sand	0.0139 [48]	10
Water	0.001 [49]	0.5
Black tea extract	0.0150	0.078

## Data Availability

Not applicable.
